# Refining empiric subgroups of pediatric sepsis using machine-learning techniques on observational data

**DOI:** 10.3389/fped.2023.1035576

**Published:** 2023-01-30

**Authors:** Yidi Qin, Rebecca I. Caldino Bohn, Aditya Sriram, Kate F. Kernan, Joseph A. Carcillo, Soyeon Kim, Hyun Jung Park

**Affiliations:** ^1^ Department of Human Genetics, School of Public Health, University of Pittsburgh, Pittsburgh, PA, United States; ^2^ Division of Pediatric Critical Care Medicine, Department of Critical Care Medicine, UPMC Children’s Hospital of Pittsburgh, Pittsburgh, PA, United States; ^3^ Division of Pediatric Pulmonary Medicine, UPMC Children’s Hospital of Pittsburgh, University of Pittsburgh, Pittsburgh, PA, United States; ^4^ Department of Pediatrics, School of Medicine, University of Pittsburgh, Pittsburgh, PA, United States

**Keywords:** biomarker, machine-learning, pediatric sepsis, observational data, clustering

## Abstract

Sepsis contributes to 1 of every 5 deaths globally with 3 million per year occurring in children. To improve clinical outcomes in pediatric sepsis, it is critical to avoid “one-size-fits-all” approaches and to employ a precision medicine approach. To advance a precision medicine approach to pediatric sepsis treatments, this review provides a summary of two phenotyping strategies, empiric and machine-learning-based phenotyping based on multifaceted data underlying the complex pediatric sepsis pathobiology. Although empiric and machine-learning-based phenotypes help clinicians accelerate the diagnosis and treatments, neither empiric nor machine-learning-based phenotypes fully encapsulate all aspects of pediatric sepsis heterogeneity. To facilitate accurate delineations of pediatric sepsis phenotypes for precision medicine approach, methodological steps and challenges are further highlighted.

## Introduction

Sepsis contributes to 1 of every 5 deaths globally with 3 million per year occurring in children. Current treatment strategies for pediatric sepsis show room for improvement. Empiric antibiotic therapies and organ-supportive treatment are employed to control infection, stabilize hemodynamics, and modulate the septic response (1). Since current septic deaths occur in children independently from the timely administration of antibiotics, ongoing pathobiological mechanisms may be at play. While the sepsis pathobiological mechanism generally involves a dysregulated immune response to infection leading to organ dysfunction (2), children with sepsis show substantial heterogeneity in various parts of the pathobiological process.

Thus, to elicit desired success in treatments and avoid “one-size-fits-all” approaches, employing a precision medicine approach is highlighted as a solution. A precision medicine approach usually develops customized plans for disease prevention, diagnosis, and treatment, for patient subgroups delineated based on input data. To delineate patient subgroups for a precision medicine approach, multifaceted sources of the heterogeneity need to be considered, including but not limited to infection etiologies, cytokine interactions, host comorbidity pattern, the timeline and characteristics of diagnosis and treatment, and host genetics (3, 4) as they were shown to impact both the disease evolution in and intervention response of septic children. This subgrouping approach has been utilized in the advanced treatment of well-studied heterogeneous diseases, such as cancer (5), but it hasn't been applied to children with sepsis. This review paper can shed light on advancing our understanding of the complex sepsis pathology and eliciting desired success in treatments.

This review provides a summary of two approaches, empiric and machine-learning-based phenotyping of pediatric sepsis based on multifaceted data. In empiric phenotyping, we will discuss pediatric sepsis heterogeneity in biological processes and clinical parameters in age- and (geographical) region-specific fashion. We will also review biomarkers that effectively stratify children with sepsis for various clinical purposes. Then, we review pediatric sepsis phenotypes delineated using machine learning techniques. Finally, realizing that neither empiric nor machine-learning-based phenotypes fully encapsulate all aspects of pediatric sepsis heterogeneity, we highlight methodological steps and challenges to facilitate further accurate delineations of pediatric sepsis phenotypes.

### Age-dependency of pediatric sepsis heterogeneity

To effectively diagnose, treat, and prevent pediatric sepsis, it is critical to understand the pathobiological processes that uniquely affect pediatric patients. Below, we will discuss the age-specificity that affects the diagnosis, treatment recommendation, risk, and underlying medical conditions (6).

To diagnose pediatric sepsis, the Pediatric Sepsis Consensus Congress (PSCC) proposed specialized medical guidelines different from adults (SEPSIS-3). These guidelines emphasize the age-specific classification for diagnosis due to different physiological and pathological processes affecting children of different ages. For example, the respiratory rate in breaths/min has a wide variability between children from 0 days to 1 week (>50), 1 week to 1 month (>40), 1 month to 1 year (>34), 2 to 5 years (>22), 6 to 12 years (>18), and 13 to 18 years old (>14) (7). As one of the most common manifestations of sepsis is increased respiratory rate, the age-dependent basal level should be considered in diagnosis. The age-dependent pathology also impacts treatment outcomes and influences recommendations for children of different ages. For example, treatments have different likelihoods of favorable outcomes from extracorporeal membrane oxygenation (ECMO) depending on the patient's age. ECMO has been shown to increase survival in cases of respiratory failure with a different survival rate between children (4 weeks to 18 years) and newborns (<4 weeks) (39% and 73%, respectively) (8).

Additionally, pediatric sepsis has risk factors that do not apply to adult sepsis or children of different ages. For example, although premature birth or low body weight are risk factors to develop severe sepsis in infants (6, 9), these risk factors would not work directly for adults nor adolescents. Lastly, pediatric sepsis demonstrates age-specificity in the underlying medical conditions. Specifically, a study showed that infants with sepsis have chronic lung disease (16.5%) and congenital heart disease (15%) predominantly. On the other hand, children from 1 to 5 years and from 5 to 9 years old commonly have neuromuscular diseases (19.5% and 24.3% respectively) while adolescents have neoplastic disorders like pre-existing cancer (from 10 to 14 years of age 23.4% and 13.8% for children 15 to 19 years old) as underlying medical conditions (9). These comorbidities result in differences in the outcome depending on the patient's age. In pediatric patients younger than 1-year-old, cardiovascular conditions and multiple organ dysfunction increase mortality with an odds ratio (OR) of 1.4 when compared to 10 to 19 years old children (10).

### Regional heterogeneity of pediatric sepsis

Pediatric severe sepsis and septic shock incidence and mortality vary depending on the combination of geographical region and ethnicity. In the USA, the incidence of severe sepsis in 2005 was 7.7% (10). In comparison, the prevalence of pediatric severe sepsis is 22.1% in Canada (11). In the SPROUT study that looks at different regions around the world, they saw a wide range in prevalence of severe sepsis. For example, 7.7%, 6.2%, 15.3%, and 16.3% of prevalence were reported in North America, Europe, Asia, and South America, respectively (12).

Furthermore, severe sepsis and septic shock had a diverse range in mortality in pediatric intensive care units (PICU) in different countries. For example, in developed countries like Italy and Japan, their mortality rate was 15% and 19% respectively (13, 14). However, in developing countries such as Brazil (15), China (16), and Colombia (17) mortality was higher (19.1%, 70%, and 34%, respectively) for septic shock.

Clinical causes underlying mortality also show regional similarities and differences between developed and developing countries. Relaxed or strict adherence to treatment guidelines has a significant impact on mortality rates both in developed and developing countries. A study showed that pediatric septic shock patients had a decrease in mortality from 38% to 6% when appropriate treatment (fluid resuscitation and inotropic therapy) was administered in hospital settings in the United States of America (18) and Brazil (19, 20). Furthermore, in developed countries, the patient's characteristics, such as the presence of immunological chronic diseases, and the characteristics of the infectious agent like the type of infectious agent are associated with higher mortality from septic shock (20, 21). For example, a retrospective study done in the USA found that previously healthy pediatric sepsis patients had in-hospital mortality of 0.7%, while patients that had an underlying medical condition had an in-hospital mortality rate of 5.1% (22). On the other hand, in developing countries, in addition to the above-mentioned risk factors, poverty, malnutrition, low vaccination rates, poor sanitary conditions, and characteristics of the health system, such as the decentralization of care, the difficulty of access to health services and the shortage of hospital beds are important factors associated with mortality (21). This can be illustrated by a study done at PICU in Brazil that showed an increase in mortality associated with unknown vaccination status of pediatric sepsis patients with a relative risk per point increase of 2.57 (23).

### Biomarker risk stratification panels for mortality in pediatric sepsis

Several phenotypes have been identified based on prognostic biomarkers that are empirically selected (Table 1) (24). Prognostic biomarkers provide information on the likely outcome of an individual and help in establishing the intensity of treatment for the patient (25). For pediatric sepsis, the most established biomarkers include C-reactive protein, erythrocyte sedimentation rate, procalcitonin, ferritin, serum thrombomodulin, CD64, and Il-8.

**Table 1:** Biomarker risk stratification panels for mortality in pediatric sepsis.

Article (Region)	Stratification	Biomarker	Other features (number)	Sample characterization	Method	Note
Assicot M et al. 1993 (France)	Subgroup I	Procalcitonin (PCT)	Clinical history and blood culture (2)	79 children with varying degrees of infection or severe burns; PICU	Laboratory tests (assays and HPLC)	A positive correlation of procalcitonin with severity of infection. Up to 200 ng/ml in patients with septic shock and Antibiotic treatment lowers procalcitonin rapidly to normal levels.
	Subgroup II					
	Subgroup III					
Ramos Garcia et al. 2007 (Brazil)		Ferritin	Sex, age, nutrition level, mechanical ventilation, days out of PICU in the 28 days after admission, PRISM, PELOD, blood culture, WBC count, alanine transaminase, aspartate transaminase, CRP (12)	36 patients with severe sepsis and septic shock; < 15 years old; PICU	Logistic regression analysis	A concentration of serum ferritin >500 ng/ml is associated with a 3.2 relative risk of death. Sensitivity: 64% Specificity: 80%
Lin JJ et al. 2017 (Taiwan)		Serum Thrombomodulin	Age, gender, site of infection, laboratory findings, mechanical ventilation, vasopressor use, severity of disease on day1, PRISM-III, PELOD, *P*-MOD, stay in PICU and hospitalization days, mortality DIC scores (14)	42 previously healthy patients with sepsis; PICU	AUC-ROC	Concentration on day 1 of diagnosis of sepsis can predict development of septic shock (AUC = 0.867, 4.71 mU/ml) and mortality (AUC = 0.863, 5.95 mU/ml).
Wong et al. 2008 (USA)	IL-8 < 220 pg/ml	IL-8	Age, sex, adverse events days, death, PRISM (4)	pediatric septic shock databases:GPSS (<10 years) and RESOLVE (<18 years); 278 patients with septic shock	AUC-ROC, statistical analysis (Student's T test, Fisher exact test, chi-square test, contingency tables)	A serum IL-8 (>220 pg/ml) predicts 28-day mortality. Sensitivity: 82% Specificity: 51%
	IL-8 > 220 pg/ml					
Tonial et al. 2021 (Brazil)		CRP, ferritin, lactate, PIM2	24 h leukocytes, age, sex, weight, type of patient, BMI, 72 h readmission to PICU, site of infection, type of infection, length of hospital stay, length of PICU stay, complete blood count, blood transfusion, mechanical ventilation, vasoactive drugs, ventilator-free days, vasoactive drug-free days, presence of complications, anemia, iron-deficiency anemia, comorbidities, death (22)	294 septic patients; <19 years >1 month; PICU	AUC-ROC, Kaplan-Meier survival curves	Alone they were associated with mortality, cutoff PIM2 (> 14%), ferritin (>135 ng/ml), and CRP (> 6.7 mg/ml) were associated with mortality. Combination: Sensitivity: 76% Selectivity: 94.5%

Among them, C-reactive protein (CRP), erythrocyte sedimentation rate, procalcitonin, and ferritin are the most widely used biomarkers in clinical settings because they are inexpensive and are already used in PICUs in many countries around the world (26–28). CRP is an acute phase reactant produced in response to cellular injury during the inflammatory response and is used as a marker of acute inflammation. When used in combination with IL-6, CRP is a reliable marker of early infection and disease progression (29). Interleukin-6 (IL-6) is a pro-inflammatory cytokine that is an integral part of the cytokine activation inflammation cascade (30). IL-6 rises fast but has a short half-life, so CRP is used to monitor disease after the 24-hour mark (29). CRP plasma levels increase within 4–6 h after the initial tissue injury and continue to increase several hundred times within 24–48 h (31). CRP remains elevated in the early stages of response and returns to normal when the damage has been managed. Thus, a rapid decrease in CRP levels over the first 48 h of therapy correlates with an effective response to the initial antimicrobial therapy in septic patients (32). Erythrocyte sedimentation rate (ESR) is used to differentiate between degrees of severity in states of inflammation. ESR has high sensitivity and specificity in the detection of inflammatory diseases and malignancy (33), however, it is not reliable in newborns since ESR sedimentation is reduced in newborns caused by a high hematocrit value (34, 35). Procalcitonin (PCT) is a precursor of the hormone calcitonin and is a reliable prognostic marker for sepsis differentiating inflammatory responses from bacterial infections. During an infection, PCT is released up to a thousand-fold increase in nearly all tissues and cell types in the host in response to cytokines and bacterial products (36). In patients with bacteremia, PCT levels are significantly higher than the patients with fungemia who have a moderate increase in PCT levels (37). The sensitivity and specificity to discriminate infection from the inflammatory response have been also reported in pediatric patients (38). Since PCT increases as disease severity worsens and falls rapidly when the infection gets controlled, it can predict sepsis mortality (39, 40). Similarly, ferritin has emerged as an important diagnostic biomarker for pediatric sepsis. High levels of ferritin are associated with poorer outcomes including death and can help differentiate between the severity stages of pediatric sepsis (41). Patients with high ferritin (≥1,000 ng/ml) were also more likely to have multiple DNA viruses detected in plasma and increased mortality (odd ratio 2.6) in pediatric severe sepsis (42), and the same association with mortality was reported by (43).

Other biomarkers, such as serum thrombomodulin have been established for adult sepsis but recently demonstrated a prognostic value for pediatric sepsis. Serum thrombomodulin levels can be used as an early predictor of mortality and disease severity in pediatric sepsis patients. In fact, when measuring pediatric sepsis patients on day 1 and day 3 of admission, non-survivors likely have higher levels of serum thrombomodulin compared to survivors. Furthermore, serum thrombomodulin levels on day 1 are strongly correlated with disease severity and can be used to predict the development of septic shock, disseminated intravascular coagulation (DIC), and multiple organ dysfunction syndrome (MODS) (44). In this prospective study, researchers analyzed the area under the curve from the receiver operating characteristic (ROC) analysis to validate serum thrombomodulin as a pediatric biomarker of sepsis using blood samples recollected from previously healthy children with sepsis, severe sepsis, and septic shock from PICU's (44).

Since sepsis is a dysregulated immune response often to infection, biomarkers for immune regulation, such as CD64 and IL-8, have also been used for prognose pediatric sepsis. CD64 is a high-affinity immunoglobulin Fc gamma receptor I and is expressed constitutively on monocytes, but only to a very low extent on resting polymorphonuclear cells (PMNs). During an infection, CD64 expression on PMNs increases to promote phagocytosis. In this regard, CD64 can be used for differentiating bacterial infection from other inflammatory disorders in children because there's an important elevation of CD64 on neutrophils in response to bacterial infection (45, 46). Also, Interleukin-8 (IL-8) is an inflammatory cytokine that is released from monocytes, endothelial cells, and neutrophils in response to IL-1 and TNF- *α*. Increases in circulating IL-8 are seen early in the infectious course and can be used as a prognostic biomarker that an elevation in IL-8 would correlate with more severe disease and mortality (47, 48).

Combining biomarkers often enhances the predictive power in complex diseases (49). A recent study demonstrates this by comparing single-biomarker models like ferritin, lactate, leucocyte count, Pediatric index of mortality 2 (PIM2), and CRP levels which in ROC analysis could predict only up to 38.6% (PIM2 alone) and a multiple-marker model (ferritin, lactate, CRP levels, and PIM2) could predict 76% of mortality with an accuracy of 0.945 (26). The Pediatric Sepsis Biomarker Risk Model (PERSEVERE) is an advanced combinatorial approach that stratifies pediatric septic shock risk based on high-dimensional biomarkers. It predicts 28-day all-cause mortality using a Classification and Regression Tree (CART) methodology on the five best-performing biomarkers identified in a transcriptomic expression study, C-C chemokine ligand 3 (CCL3), heat shock protein 70 kDa 1B (HSPA1B), interleukin 8 (IL8), neutrophil elastase 2 (ELA2), and lipocalin 2 (LCN2). When applied to a test cohort, sensitivity was 89% and specificity was 64% (50). Since the CART model is flexible to integrate variables of different natures, it has been refined and updated (51) in additional studies since its inception. For example, PERSEVERE-II added platelet count (52) and PERSEVERE-XP included four mRNA biomarkers (protein regulator of cytokinesis 1 (*PRC1*), histidine ammonia-lyase (*HAL*), DNA damage-inducible transcript 4 (*DDIT4*), and ZW10 interacting kinetochore protein (*ZWINT*) (53) and achieved further improvements. For example, PERSEVERE-XP had a 95% sensitivity and 81% specificity for detection of pediatric sepsis mortality which was superior to 81% sensitivity and 74% specificity from PERSEVERE alone (53).

### Empiric phenotypes of pediatric sepsis

In a related approach based on clinical observations combined with a limited set of key biomarkers, Carcillo J,2019, specified 4 overlapping phenotypes of multiple organ failure patterns in pediatric sepsis, Thrombocytopenia associated MOF (TAMOF), Sequential liver failure associated MOF (SMOF), Immunoparalysis associated MOF (IPMOF), and Macrophage Activation Syndrome (MAS) based on physiologic, clinical and biomarkers variables such as specific organ failure patterns, platelet count, soluble Fas ligand, whole blood ex vivo TNF-α response to endotoxin, ADAMTS13 activity, and Ferritin (Table 2). TAMOF is characterized by thrombotic microangiopathy with reduced ADAMTS13, SMOF has elevated levels of sFasL. IPMOF is defined by prolonged immunodepression, and MAS has uncontrolled inflammation. All these phenotypes have varied prevalence and mortality rates among patients. SMOF, TAMOF, and MAS phenotypes were associated with higher mortality (around 45% in SMOF, TAMOF, and MAS vs. 20% in IPMOF) and clinical trials have started to assess if personalized treatment for these phenotypes leads to better outcomes (54). Similarly, Xiang et al., 2021 proposed the pediatric sepsis-induced coagulation score (pSIC), which classifies immune-dysregulated pediatric sepsis patients based on prothrombin time, platelet count, and pediatric Sequential Organ Failure Assessment (SOFA) score derived from 4 items (respiratory SOFA, cardiovascular SOFA, hepatic SOFA, and renal SOFA). pSIC scores the degree of sepsis-induced coagulopathy (SIC) for pediatric patients based on age-related pathophysiological and clinical differences. Using Kaplan–Meier survival curve analysis, they found patients with a high pSIC score (pSIC ≥ 4) have worse clinical outcomes than the non-pSIC group (pSIC < 4) with 0.716 in the area under the curve of ROC for predicting 28-day mortality (55).

**Table 2:** Empirical and machine-learning-based phenotypes of pediatric sepsis.

Article (Region)	Stratification	Features	Sample characterization	Method	Note
Wong et al. 2009 (US)	A, B, C	whole-genome Expression profiling	98 children ≤10 years old	Hierarchical clustering	
Sanchez-pinto et al., 2020 (US)	“Severe, persistent encephalopathy”, ‘Moderate, resolving hypoxemia’, ‘Severe, persistent hypoxemia and shock’, ‘Moderate, persistent thrombocytopenia and shock’	6 clinical features (pSOFA subscore) across multiple PICU days	5,054 children ≤21 years old	subgraph-augmented nonnegative matrix factorization	This study collected patients with MODS
Carcillo et al., 2021 (US)	TAMOF, SMOF, IPMOF	5 clinical features and 3 biomarkers	401 children ≤18 years old	Empirical method	
Xiang et al., 2021 (China)	pSIC group, non-pSIC group	pediatric sepsis-induced coagulation (pSIC) score induced by 3 clinical features	91 non-premature infants and children ≤18 years old	Empirical method	predict 28-day mortality in sepsis was, with the cutoff value of > 3 (0.716 AU-ROC)
Koutroulis et al., 2022 (US)	Phenotype 1,2,3,4	22 clinical features or 29 Clinical features	151 non-neonatal children	Latent class analysis, K-means clustering	22 clinical features were selected from William et al.'s study and 29 clinical features were selected from Seymour et al.'s study.
Qin et al., 2022 (US)	PedSep-A, B, C, D	23 Clinical features and 2 biomarkers	404 children ≤18 years old	Consensus K-means clustering	

### Machine-learning-based subgroups of pediatric sepsis

Although these empiric phenotypes successfully stratified children with sepsis for particular clinical purposes, they are not designed to fully appreciate the innate heterogeneity of pediatric sepsis. This limitation led to the desire to refine phenotypes using machine-learning (ML) techniques. ML allows computers to agnostically discover patterns in the data, without being given a set of explicit instructions (56) (Table 2). Wong et al. (2009) used transcriptomic data and found and validated three subgroups in septic shock pediatric patients, subclass A, B, and C, through discovery-oriented expression filters and unsupervised hierarchical clustering. Subclass A had the highest pediatric risk of mortality (PRISM) III score, degree of organ failure, mortality rate (36%), and significant differences in gene expression patterns in pathways related to the adaptive immune system and glucocorticoid receptor signaling that could be studied more to identify therapeutic targets. The PRISM score is one of the widely used scoring systems to quantify critical illness in the pediatric age group. On the other hand, subclass B and C did not present predominantly characteristic features (57, 58).

In another study conducted by Sanchez-Pinto and colleagues, the subgraph augmented nonnegative matrix factorization method revealed 4 distinct phenotypes in 5,054 critically ill pediatric patients with MODS) (59). Among these phenotypes, Phenotype 1 was characterized by severe and persistent encephalopathy. Phenotype 2 was characterized by moderate and resolving hypoxemia. Phenotype 3 was characterized by severe, persistent hypoxemia and shock, and Phenotype 4 was characterized by moderate, persistent thrombocytopenia and shock.

Koutroulis et al., (2022) performed a systematic analysis using latent class analysis (LCA), a mixture model that detects latent (or unobserved) heterogeneity in data, on 151 pediatric sepsis patients. To make the clusters they used variables that had been used in other studies, which are 22 variables based on PRISM score (61) or 29 variables based on sepsis onset (62). The most important characteristic of the 4 phenotypes delineated from the first data set was Phenotype 1, which is characterized by multiple organ dysfunction. On the other hand, Phenotype 2 is characterized by low severity with only a few elevated WBC parameters, phenotype 3 shows moderate severity with mild tachypnea, and phenotype 4 presents high severity with liver dysfunction with hypoxia. These 4 phenotypes were found to match well with the phenotypes from the other data set, demonstrating moderate reproducibility (60).

In a recently published study, our research group used the consensus k-means clustering analysis of 25 available bedside variables including C-reactive protein and Ferritin levels at 24 h to identify 4 phenotypes in severe sepsis patients with organ failure (63). PedSep-A is defined by younger children (mean of 3 years) with respiratory failure, with a low (2%) mortality (2%), PedSep-B is characterized by multiple organ failures with requirement for intubation with a medium (12%) mortality, PedSep-C had cardiovascular failure, lymphopenia and high ferritin with a medium (10%) mortality, and PedSep-D is distinguished by multiple organ dysfunction and a high mortality rate (36%).

**Figure 1:**
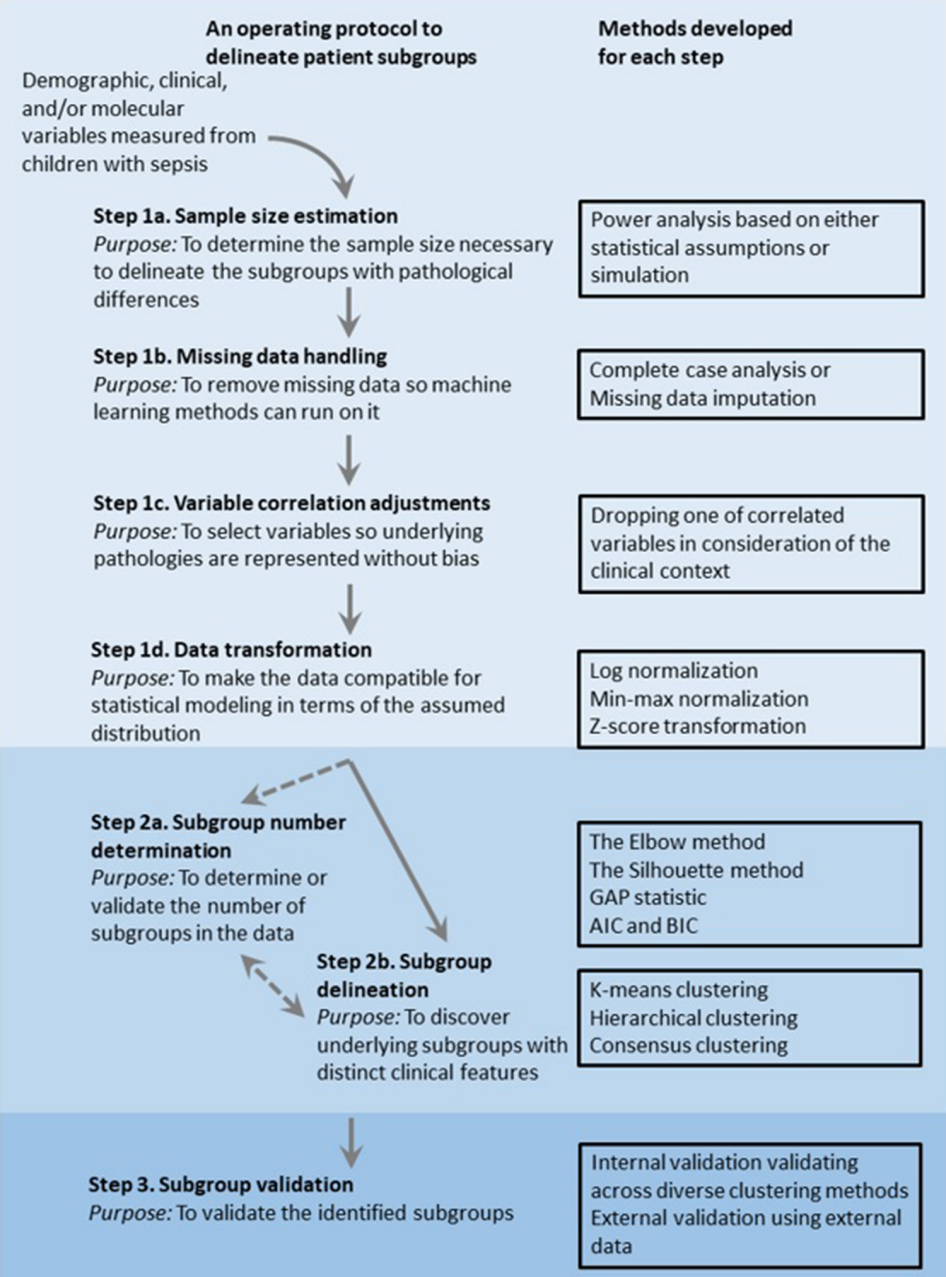
Overview of an operating protocol to delineate patient subgroups using observational data of clinical features.

However, these phenotypes of pediatric sepsis still do not consider all the factors implicated in the age- and region-specificity nor potentially complex effects of nutrition, vaccination, and treatment strategy underlying the region-specificity. To guide further efforts of machine-learning-based phenotyping, we will provide general algorithmic steps applicable to delineate pediatric sepsis subgroups below (Figure 1).

### Preprocessing of clinical data for machine-learning-phenotypes

ML allows computers to agnostically discover patterns in the data and improve with experience, without being given a set of explicit instructions. Since it does the job without a set of instructions, it is expected to create nonsynonymous patient subsets by mining the clinical data in an unbiased fashion to improve the phenotype delineations from empiric approaches. Below, we will discuss several challenges to successfully develop machine-learning methods to develop agnostic phenotypes for pediatric sepsis.

### Sample size examination

Small sample-size and single-center studies are more common in studies of pediatric sepsis due to the ten times less common prevalence of sepsis in children compared to adults (64). A meta-analysis conducted by Menon et al. indicated that some pediatric sepsis studies were conducted with a very small sample size (i.e., less than 50) (65). With this limited sample size, the chance to delineate the phenotypes is low. In statistics, this chance is formally estimated by statistical power which indicates the probability of rejecting the null hypothesis that no phenotypes exist in the data. Therefore, power analysis is necessary for the data collection step to quantitatively determine the smallest sample size for detecting the phenotypes at the desired level of significance.

The simplest approach to conducting power analysis is standard power equation, where powers can be directly estimated given a fixed set of input parameters, such as expected effect size and standard deviation in the population (66). However, standard power equations are based on few assumptions of input data, so they are not suitable in practical studies with complex data structures and flexible study design (67). To address this problem, researchers can perform simulation experiments where datasets are repetitively generated with respect to the statistical properties of input data to calculate the proportion of experiments in which the null hypothesis is rejected (68). With the aid of simulation experiments, investigators can estimate power more precisely by taking real data properties into account. For example, Koutroulis et al., 2022 and (61, 62) estimated the smallest sample size to delineate phenotypes through simulation experiments. As a result, both found that 150 samples were enough to identify 4 distinct phenotypes with 80% power (60).

### Missing data handling

Clinical data can be missing for several reasons, such as (i) physicians not ordering certain laboratory measures (e.g., cholesterol test not ordered for all patients); (ii) mechanical error (e.g., sphygmomanometer failure); and (iii) patient refusal to respond to questions (e.g., income-related questions). Missing data can prohibit a successful delineation of phenotypes, as most machine learning algorithms, including phenotyping methods, assume the data completeness (69). Two commonly employed strategies to handle missing data are complete case analysis and missing data imputing. The complete case analysis discards the observations with missing data whereas the missing data imputation replaces the missing data with estimations (70). Although the complete case analysis is much simpler than imputation-based approaches, it can cause a significant loss of samples. This can aggravate the sample size issue, especially in small-scale studies. Further, if there was a relationship between missing data and the values of variables in the data (missing data mechanism), then this approach can introduce bias. Thus, the imputation strategy serves as a reasonable alternative to complete case analysis.

Several imputation methods are widely adopted in the field. Choosing the proper method requires a deep insight into the missing mechanisms. Systematically, missingness can be categorized into three patterns according to the missing mechanism: (1) missing completely at random (MCAR), (2) missing at random (MAR), and (3) missing not at random (MNAR) (70). MCAR holds when data are missing by mere accident or study design, which is usually not likely in practical scenarios. MAR is more common in practical scenarios. It occurs when data are missing through known mechanisms related to patient characteristics, where the likelihood of one variable's missingness does not depend on the variable itself but is conditional on other covariates with full information in the data set (70). Hence, although observed values of a variable differ systematically from missing values, methods such as Multiple Imputation can use other observed covariate data to correct the differences and perform imputation appropriately (71). Contradicted to MAR, MNAR holds when missing data are systematically different from observed values for unknown reasons so that there is no way to utilize the information of observed data (70). Thus, missingness should be modeled explicitly with a proper method. For the case of outcomes with MNAR-based missingness and covariates with MAR-based missingness, the Heckman imputation model has been proposed to impute the missing data by using a method named MICE (Multiple Imputation by Chained Equations) (72). Furthermore, investigators should also be aware that when the missing rate is exceptionally high in some variables, removing the variables with high missingness may serve as a reasonable strategy to ensure better performance of imputation.

### Correlation adjustment

Correlation measures the degree to which two features relate to each other. In observation data collected from children with sepsis, significant correlations can be observed among cytokines and routine laboratory data (73). If one wants to incorporate a linear-model-based machine learning method, this would yield multicollinearity, which makes it difficult to estimate the effect size accurately. A phenotyping analysis can use the correlation relationship to control the bias due to different weights of biological mechanisms considered. For instance, both C-reactive protein (CRP) and erythrocyte sedimentation rate (ESR) represent inflammation in pediatric sepsis pathology, thus they would be highly correlated (74). In this case, by considering both phenotypes, a subtype analysis can overestimate the effect size of the inflammation process and therefore introduce a bias toward inflammation. To reduce the bias, one can identify the correlated feature pairs and remove one of the features to represent underlying factors equally (75). To determine which feature in the correlated feature pairs to remove, the missingness and the clinical context of the variables can be considered. For example, if two clinical variables that show a correlation in the data have different missing data ratios, investigations can first consider dropping the feature with higher missingness. If the two variables have similar missingness, their clinical context can be further considered. For example, if CRP and ESR are correlated and show similar missingness in a study investigating a time-sensitive aspect of sepsis, keeping CRP and removing ESR would make more sense since CRP is a more sensitive indicator of rapid inflammatory response than ESR (74).

### Data transformation

To build a statistical model using clinical data, a critical problem is that clinical data usually do not follow normal (Gaussian) distribution, while most statistical models assume normal distribution. To address this problem, investigators need to evaluate if variables in the data follow normal distribution. And the variables not following normal distribution need to be transformed towards a more normal distribution before being fed to statistical models. Specifically, to determine if a variable follows normal distribution, graphical tools such as histogram, box plot, and quantile-quantile plot can be used to show it the data comes from the normal distribution. Other statistical tests of normality include Shapiro-Wilk (75), Kolmogorov–Smirnov (76, 77), Lilliefors, and Anderson–Darling tests (78). Among them, Shapiro-Wilk test is the most powerful test through examination based on simulation analysis (79). Then, to transform those that do not follow normal distribution, there are multiple transformation methods to address different aspects of the problem. First, clinical data values are often highly skewed due to extreme clinical cases, such as severe sepsis and septic shock. Since the skewness shows how much data is asymmetrical from the normal distribution, it will introduce bias in the statistical estimation that assumes the normal distribution. To normalize a highly skewed variable, log-transform is one of the most frequently applied approaches (80). Second, another essential transformation is data-scaling when the range of clinical variables varies extensively. Without this step, features with a broader scale range can overwhelm the downstream statistical process, including machine-learning-based phenotyping. Broadly accepted solutions to this problem include min-max normalization and *Z*-score normalization. Under min-max normalization, the minimum value of each feature gets transformed into a 0, the maximum value gets transformed into a 1, and every other value gets transformed into a decimal between 0 and 1 accordingly. *Z*-score normalization transforms the values such that the mean of all of the values is 0 and the standard deviation is 1. The normalization schemes come in different pros and cons when used for multiple variables. For example, although min-max normalization puts all variables on the same exact scale, it does not handle outliers well. And although *Z*-score normalization handles outliers by diluting the effect in the condition that the mean is 0 and the standard deviation is 1, it does not put variables on the same scale.

### Delineating and validating phenotypes

After the preprocessing steps, multiple machine learning methods can be used to further delineate and validate phenotypes. To select the right method for the data at hand and make appropriate interpretations from the results, it is important to understand the pros and cons of the methods, which we will discuss below.

### Unsupervised learning of the underlying clusters for phenotyping

Unsupervised learning methods generally refer to statistical approaches that learn the parameters of the underlying model without first training by labeled data. Since the training based on labels may serve as bias, unsupervised learning methods recently gained popularity to delineate phenotypes of complex diseases in an unbiased fashion. When the number of phenotypes is relatively clear, *K*-means clustering is widely used (81) due to its computational simplicity and interpretability. On the variable space where observations are placed as data points with respect to the variable values, it initially selects a particular number (*K*) of random points as cluster centers (i.e., centroid) and assigns the data points to the closest centroid based on a distance metric. In low-dimensional data, Euclidean distance, which calculates the root of square difference on the variable space between object pairs, is widely used if the feature values are continuous. If the data has many variables and makes a high-dimensional variable space, Manhattan distance can be used to control distributional discrepancy between variables. Also, if the data have categorical variables, which is often the case in clinical data (e.g., comorbidities, symptoms), Hamming distance can be used for its flexibility to handle different numbers of category values. After the first assignment with respect to the centroids, the algorithm repeatedly updates the centroids and the assignments until the intra-cluster variation is minimized (i.e., convergence) (81).

Although K-means clustering is an unsupervised clustering, it still requires prior knowledge about the number of clusters in the data. If prior knowledge regarding the number of clusters is not available, one can use another well-established method called hierarchical clustering. Hierarchical clustering can be further categorized into agglomerative and divisive clustering (82). The agglomerative algorithm first considers each object as a single cluster and iteratively combines the most similar leaf pairs into a larger cluster. Oppositely, the divisive algorithm starts with one large cluster that has all objects and recursively splits it into smaller clusters. Principally, both K-means and hierarchical clustering methods can be iteratively performed on subsampled data to obtain a consensus clustering assessment (83). Due to the robustness, the consensus clustering approach, either based on K-means or hierarchical clustering, has been well accepted and successfully applied in several critical care studies (Seymour et al*.*, 2019; Soussi et al*.*, 2022) (62, 84). To validate the clustering result, methods of different approaches can be used to ensure generalizability. For this purpose, algorithms based on mixture models are widely used, such as latent class analysis (LCA) and latent profile analysis (LPA) (85). Due to differences in approach, the mixture model-based methods uniquely provide a probability distribution over the cluster assignment for each object (86). With the distribution estimation, the mixture models allow more flexibility in cluster membership determination without clear-cut assignment. However, by the same token, this approach does not guarantee to assign all input samples to a subgroup.

### Cluster number determination

Determining the optimal cluster number is a fundamental step for unsupervised clustering methods to either as an input to certain clustering algorithms (e.g., *K*-means) or to validate the cluster numbers identified in other approaches. There have been several methods proposed to determine the optimal cluster number. One of the earliest proposed and most popular methods is the Elbow method (87). Given a preset range of the cluster numbers, the Elbow method employs an external clustering method with each cluster number. Then it calculates the sum of squared errors (SSE) for each specified cluster number and plots a curve of SSE against the number of clusters. Finally, it defines the “elbow” of the curve with the most dramatic change of the curve as the optimal cluster number. This is based on the rationale that, although adding more clusters does not hurt the fit and explains more of the variation, the improvement becomes not worth the added complexity brought by the clusters at some point (88). Adding more clusters beyond the elbow point often means clustering for the noise or data-specific signals of the data, also known as an over-fitting problem. However, the Elbow method becomes unreliable when the SSE plot is fairly smooth and the elbow point is hard to unambiguously distinguish (89). Also, the Elbow method only employs Euclidean distance to evaluate the improvement and is thus appropriate for datasets with small size and low complexity.

If the data has many samples and represents a complex pathology, one can use the Silhouette method. The Silhouette method considers the variable distribution in the form of variance, skewness, high-low differences, etc. to quantify the tightness and separation of objects within the assigned cluster by a value ranging from 1 to −1. A higher positive value implies a better matching of an object to its cluster, whereas a lower negative value denotes a poorer matching performance. Therefore, the Silhouette method also can be used to evaluate clustering performance (90).

Another commonly adopted cluster number determination method is gap statistic, which compares the change in observed within-cluster dispersion with an expected dispersion under a simulated null reference distribution of the data, i.e., a distribution with no obvious clustering (91). Since the gap statistic measures the difference from the null distribution, the clustering number that maximizes the gap statistic would be optimal. However, to handle real-world datasets where the clusters are not as well-defined, the maximization needs to be balanced with the added complexity brought by the clusters. To evaluate the balance, one can conduct a simulation study with various numbers of clusters and choose the cluster number that makes the difference from the null distribution by more than the simulation error.

### Phenotype validation

After delineating a set of phenotypes for pediatric sepsis, researchers should evaluate the validity of subtypes through internal validation, external validation, and clinical plausibility determination. Regarding internal validation, several evaluation metrics can be employed to measure the robustness of the phenotypes. As mentioned in the cluster number determination section, Silhouette is one of the widely adopted intrinsic cluster quality measures that does not require ground truth labels (91). Another internal validation strategies involve comparing the cluster similarity across various clustering methods. Although various methods are designed under different assumptions and thus can lead to disagreement in the assignment, valid cluster memberships ought to be similar across diverse clustering methods. Additional to internal validation, external validation assesses the reproducibility of clustering by utilizing an extra source of data. Normally, investigators use external datasets collected with the same criteria as the exploratory dataset and perform the same analysis to see if clusters with similar clinical characteristics can be discovered. Finally, it is of great significance to examine the clinical plausibility of derived phenotypes. Multiple types of variables other than those already used in clustering (e.g., bedside records. laboratory data, biological data, outcome information, a therapeutic response) can be collected from the same cohort to investigate the phenotype diversity (63) under the rationale that the phenotypes are expected to be reflected in the additional variable set (92).

## Discussion

In this manuscript, we reviewed pediatric sepsis heterogeneity reflected in the age- and region-specificity. Then, we discussed two main approaches for phenotyping septic children with less heterogeneity based on the clinical characteristics, using empiric and machine-learning approaches. First, we explored the biomarkers that have been developed to empirically subgroup the heterogenous septic children for particular clinical purposes, e.g., to diagnose early infection and disease progression or to predict deadly outcomes. For machine-learning-based phenotyping approaches, we laid out several phenotypes delineated using diverse data types. In the phenotype reviews, we further realized that the current phenotypes do not fully grasp the heterogeneity implicated in the age- and region-specificity. Thus, to facilitate the further development of machine-learning-based phenotyping representing the full spectrum of the heterogeneity, we discussed essential data handling steps from a statistical point of view.

Although the approaches have shown success in improving pathological understanding of complex diseases, we recognize their limitations. First, when diverse types of molecular and clinical variables (e.g., gene expression and laboratory measures, respectively) are used for this purpose, the underlying disease mechanisms may be represented across multiple variables with different weights. Since the multiple representations with different weights would result in bias in the phenotyping, using high-dimensional data for phenotyping requires careful statistical handling of the variables. To address this problem, variable selection methods or dimensionality reduction methods can be used to identify a smaller set of representative variables that would represent the underlying mechanisms with less redundancy. For example, principal component analysis (PCA) can be used to select the variables that linearly explain much of the data variance. Second, empiric and machine-learning approaches would yield different sets of patient subgroups. To reveal phenotypes with distinct identities in the data, it is not clear how to reconcile the different sets of patient subgroups identified by the two approaches. Third, subgroup memberships may not be considered distinct identities since patients may carry characteristics of multiple subgroups with various weights (63). Thus, downstream analyses may need to consider the weights, or the different confidence levels of the phenotype assignments for clinically and biologically reasonable interpretations. Since phenotyping patients of a heterogenous complex disease serves as the first step of precision medicine, we believe that this review will aid in improving clinical outcomes of pediatric sepsis.
